# Gender Differences in Anxiety Among COVID-19 Inpatients Under Isolation: A Questionnaire Survey During the First and Second Waves of the COVID-19 Pandemic in Japan

**DOI:** 10.3389/fpubh.2021.708965

**Published:** 2021-10-20

**Authors:** Ryo Tsukamoto, Yuki Kataoka, Koichi Mino, Naoki Ishibashi, Mariko Shibata, Hiroo Matsuo, Hironobu Fujiwara

**Affiliations:** ^1^Department of Psychiatry, Hyogo Prefectural Amagasaki General Medical Center, Amagasaki, Japan; ^2^Department of Internal Medicine, Kyoto Min-Iren Asukai Hospital, Kyoto, Japan; ^3^Clinical Epidemiology Section, Department of Community Medicine, Kyoto University Graduate School of Medicine, Kyoto, Japan; ^4^Department of Healthcare Epidemiology, Graduate School of Medicine and Public Health, Kyoto University, Kyoto, Japan; ^5^Department of Psychiatry, Hyogo Prefectural Hyogo Mental Health Center, Kobe, Japan; ^6^Department of Infectious Disease Internal Medicine, Hyogo Prefectural Amagasaki General Medical Center, Amagasaki, Japan; ^7^Department of Neuropsychiatry, Faculty of Medicine, Kyoto University, Kyoto, Japan; ^8^RIKEN Center for Advanced Intelligence Project, Artificial Intelligence Ethics and Society Team, Kyoto, Japan

**Keywords:** COVID-19, anxiety, gender differences, coping, isolation, Japan, mental health

## Abstract

This study assesses the gender differences in health and anxiety, especially pertaining to mental health problems and time-course effects. We surveyed 121 patients admitted to a hospital with a COVID-19 diagnosis between March 1 and August 31, 2020. Their mental status was evaluated on admission using the Japanese General Health Questionnaire-28 (GHQ-28) and State–Trait Anxiety Inventory—Form JYZ (STAI). The patients were divided into two groups depending on the period of prevalence, that is, the first and second waves of the pandemic in Japan (from the beginning of March to the end of May 2020, Time 1 = T1; and from the beginning of June to the end of August 2020, Time 2 = T2). A multivariate analysis of covariance revealed significant differences in gender by time interactions in the GHQ-28 subscale “Insomnia and anxiety” and STAI subscale “State–Anxiety.” *Post-hoc t*-tests revealed that the scores of “Insomnia and Anxiety” and “State–Anxiety” were higher in women than in men at T1. However, no difference was observed at T2. Further, “Insomnia and Anxiety” and “State–Anxiety” were significantly higher at T1 than at T2 in female patients. There was no significant difference in males. Thus, female patients were more anxious and depressed in the early phase of the pandemic, whereas male patients had difficulties in coping with anxiety. We suggest more gender-specific mental care, particularly for women at the early stages of infection.

## Introduction

The spread of the coronavirus disease (COVID-19) has been unprecedented in the history of pandemics in the twenty-first century. Since its detection in late 2019, the disease has spread worldwide and has been associated with a spate of medical emergencies and post-recovery health problems, besides being potentially fatal. Despite the lull following the first wave (*primary wave*) of COVID-19 in Japan, there were signs of a prolonged course of infectious spread and a second outbreak (*secondary wave*). Over the course of this period, experts have accumulated new and more in-depth knowledge of the disease, which has helped establish systems to respond to the pandemic.

Some early studies showed that COVID-19 seriously affects mental health (1–3), while others demonstrated that exposure to it has a minimal direct association with mental health (4). This inconsistency in the impact of COVID-19 on mental health needs to be clarified, to achieve better mental health care for COVID-19 patients. Furthermore, the mental health of COVID-19 patients can also be influenced by factors such as the duration of the pandemic and the way of information dissemination by the media. We started conducting a continuous follow-up mental health survey, including a follow-up planned for after the end of this COVID-19 pandemic, since it has not fully converged so far anywhere in the world, including Japan. Most of the research related to COVID-19 and mental health has only focused on the mental health of the general population. There are a few studies that have examined the mental health of COVID-19 patients who showed more severe symptoms including post-traumatic symptoms, depression, anxiety, and somatic symptoms than control groups (5). It has been reported that 30.2% of patients afflicted with COVID-19 had PTSD, which was more common in women (6). However, little is known about mental health problems in hospitalized inpatients afflicted with COVID-19. Therefore, it would be beneficial to present the basic data of mental health problems in hospitalized COVID-19.

Existing research also confirms gender differences regarding mental health. For example, women predominantly have internalizing symptoms, often because of depression and anxiety, while men tend to experience externalizing symptoms (i.e., violence or substance abuse) (7). Moreover, women seek emotional support to cope with stress more than men (8). As for the gender differences concerning mental health during COVID-19 pandemic, only a handful of reports were found as the period is too limited to accumulate adequate evidence. There is one report which suggests that the factors affecting anxiety and depression differed by gender in COVID-19 patients: male patients whose colleagues were also infected with COVID-19 tended to have more depression and anxiety because colleague infections mean that the workplace of participants may be in the outbreak area, which could company bankruptcy or patient unemployment. Women, however, had higher anxiety depending on their physical symptoms. They were more interested in communicating with medical staff (9). To date, to our knowledge, there is no study considering gender differences at more than one time point in patients with COVID-19. It would be worthwhile to obtain clinical knowledge that contributes to better understanding of mental support strategies, by conducting surveys with patients over time as the situation improves and deteriorates, as there is still no positive prospect of the convergence of the pandemic. Thus, we believe it necessary to investigate gender-based differences in mental health issues among patients of COVID-19. Thus, we believe it necessary to investigate gender-based differences in mental health issues among patients of COVID-19. By specifically considering the differences in patients' mental health between the primary and the second wave of the pandemic, we comparatively assessed the general health and anxiety of men and women as indices of anxiety/depression at the subclinical level. This exploration can offer gender-specific insights to practitioners caring for the mental health of COVID-19 patients. We hypothesized that women had more severe mental health problems during both the primary and secondary waves of the ongoing pandemic.

## Methods

This study was a single-center retrospective cohort study conducted at Hyogo Prefectural Amagasaki General Medical Center (AGMC), Hyogo, Japan. The number of people with COVID-19 detected in the first and second waves were about 124 and 525 per day (10), respectively. We surveyed 121 patients (mean age = 46.4 ± 17.5, men = 66, women = 55) who were admitted to the hospital after being diagnosed with COVID-19 using the real-time reverse transcription–polymerase chain reaction test between March 1 and August 31, 2020. Each patient's past medical history was recorded, and they were subsequently isolated in either a single or a quadruple room. We evaluated their mental health status on admission using the Japanese General Health Questionnaire-28 (GHQ-28), which is an instrument for estimating psychosocial well-being (11, 12), and State–Trait Anxiety Inventory–Form JYZ (STAI), which is an instrument used to estimate anxiety (13). The GHQ-28 is a bimodal scoring scale for assessing general health (the score range from 0 to 28). It consists of four subscales (physical symptoms, anxiety/insomnia, social activity, and depression tendency and seven questions for each item). The STAI is a four-point Likert scale that consists of 40 questions that assess state and trait anxiety, ranking it from 1 to 5, with the higher the anxiety, the higher the score. The Japanese version of both GHQ-28 and STAI, which are widely used, have been standardized and published as products (14, 15). The internal consistency reliability of the Japanese version of the GHQ-28 (Cronbach's alpha = 0.88) suggested a high level of reliability (16). The Pearson's correlation coefficient between the GHQ and PSE (Present State Examination) was 0.659. Furthermore, the correlation between the Japanese version of the full scale (GHQ-60) and GHQ-28 was high (*r* = 0.9695), which is indicative of the fact that the Japanese version of the questionnaire was constructed well in terms of reliability and concurrent validity. The internal consistency reliability of the STAI (Cronbach's alpha) ranged from 0.896 to 0.918 in both trait and state anxiety for both genders, with high test-retest reliability (*r* = 0.856). The correlation between the STAI and Cattle Anxiety Scale was high (*r* = 0.67). These results indicated that the Japanese version of the questionnaire was satisfactory in both its reliability and validity (13). The patients were divided into two groups, depending on the period of prevalence during which they contracted the infection: the primary (Time 1 = T1) and secondary (Time 2 = T2) waves of the pandemic in Japan, that is, from the beginning of March to the end of May 2020, and from the beginning of June to the end of August 2020, in accordance with a formal report by the National Institute of Infectious Diseases in Japan, in which the First Wave in Japan was from January 16 to May 31, and the Second Wave from June 1 to August 19 (10).

Patients received remote medical examinations from psychiatrists through a video call application (FaceTime^®^) on a tablet device. It was found that 70 patients could not use the device due to the severity of their symptoms, such as COVID-19-induced pneumonia. To prevent infection, we covered the tablet devices, which were exclusively used for the survey, with disposable plastic bags. Those who could not use the video call application on a tablet device were excluded. We determined the patients' psychological state by referring to the GHQ-28 score. This study was approved by the Hyogo Prefectural Amagasaki General Medical Center Institutional Review Board. The participants provided verbal informed consent.

Patients' demographic data were analyzed using two-tailed *t*-tests or χ^2^-tests. Between-group differences in the data were assessed using a multivariate analysis of covariance (MANCOVA), with gender and time (T1 and T2) as between-subjects factors, the scores of GHQ-28 and STAI as dependent variables, and age and presence of history as covariates. *Post-hoc t*-tests were used to compare the GHQ-28 and STAI scores at each point in time (T1 and T2) for both male and female participants, as well as for comparing the scores at T1 and T2 for each gender. Additionally, the time course changes (from T1 to T2) of these scores were investigated for each gender. Statistical significance was set at *p* < 0.05.

## Results

The summary of the demographics for this study was as follows: age was matched between male and female participants both at T1 and T2 [T1: male/female = 47.4 (16.2)/46.9 (15.3), *t* = 0.053, *p* = 0.958; T2; male/female=45.8 (18.2)/46.2 (18.4), t = 0.085, *p* = 0.932]. The number of male and female participants was also matched between T1 and T2 (T1 [male/female = 23/19], T2 [male/female = 43/36], χ^2^ [l] = 0.001, *p* = 0.972). No gender difference was found in terms of presence of medical history (male/female = 5/9, χ^2^ [l] = 2.264, *p* = 0.132), and the number of participants with medical history did not differ between T1 and T2 (T1/T2 = 6/8, χ^2^ [l] = 0.464, *p* = 0.496). The participants answered the GHQ-28 and STAI. A summary of MANCOVA is reported in Table 1. In brief, the analyses revealed a significant effect of gender on GHQ-28 scores (total score, anxiety/insomnia, and depression subscales) and STAI scores (state–anxiety, trait–anxiety). No significant effect of time was found in either GHQ-28 or STAI scores. “Gender by Time” interactions were significant in the GHQ-28 subscale of anxiety/insomnia (*F*[1] = 5.591, *p* = 0.02) and state–anxiety (*F*[1] = 7.095, *p* = 0.009). Regarding the effects of covariates on the differences of the GHQ-28 and STAI, neither age (*F*[1] = 0.742, *p* = 0.391, *F*[1] = 0.036, *p* = 0.849 and *F*[1] = 0.485, *p* = 0.487, respectively) nor the medical history (*F*[1] = 1.003, *p* = 0.319, *F*[1] = 0.045, *p* = 0.832 and *F*[1] = 3.256, *p* = 0.074, respectively) showed any effect in the analysis.

**Table 1:** Group comparisons between male and female patients.

	**Male** **(*N* = 66)**	**Female** **(*N* = 55)**	**Gender effect**		**Time effect**		**Gender–time** **interaction**	
	**M(SD)**	**M(SD)**	**F(1)**	***p***	**F(1)**	***p***	**F(1)**	***p***
**GHQ-28^a^**								
Total score	6.32 (4.77)	8.42 (5.96)	4.972	0.028^*^	1.015	0.316	1.213	0.273
Physical symptoms^b^	2.70 (2.25)	3.13 (2.36)	1.231	0.270	2.614	0.109	0.464	0.497
Insomnia and anxiety^b^	1.91 (1.86)	2.71 (2.11)	7.796	0.006^*^	1.053	0.307	5.591	0.020^*^
Social activity^c^	1.33 (1.77)	1.67 (1.97)	0.785	0.377	0.194	0.660	0.042	0.839
Depression^c^	0.32 (0.77)	0.87 (1.59)	4.420	0.038^*^	1.082	0.300	0.039	0.843
**STAI^d^**								
State–anxiety	2.70 (0.86)	3.02 (1.10)	6.354	0.013^*^	2.440	0.121	7.095	0.009^*^
Trait–anxiety	2.32 (1.00)	2.93 (1.20)	10.119	0.002^*^	0.022	0.882	2.753	0.100

*GHQ-28, General Health Questionnaire-28; STAI, State–Trait Anxiety Inventory; SD, standard deviation;*

* *p < 0.05*.

a *GHQ-28 consists of four evaluation items (physical symptoms, anxiety/insomnia, social activity, and depression tendency), and each item has seven points. A GHQ-28 total score (a perfect score of 28 points) of more than six points is suggestive of some psychological distress symptoms*.

b *A score of more than four points for the items of physical symptoms or Anxiety/Insomnia is suggestive of moderate psychological symptoms*.

c *A score of more than three points for the items of social activity or depression tendency is suggestive of moderate psychological symptoms*.

d *STAI consists of two evaluation items (State–Anxiety, Trait–Anxiety), and each item is evaluated on a scale of 1 to 5, and the higher the anxiety, the higher the score*.

*Post-hoc t*-tests revealed that the GHQ-28 total score and the anxiety/insomnia score were higher in female than male participants at T1 (t = 2.241, *p* = 0.031 and t = 3.504, *p* = 0.001, respectively) (Table 2). No gender differences were found in these items at T2 (t = 1.060, *p* = 0.292 and t = 0.407, *p* = 0.685, respectively).

**Table 2:** Group comparison by time.

	**T1^a^**		***Post-hoc* test Male/Female**	**T2^b^**		***Post-hoc* test Male/Female**	***Post-hoc*** **test between** **T1 and T2 in each gender**	
	**Male**	**Female**		**Male**	**Female**		**Male**	**Female**
	**M(SD)**	**M(SD)**	**t(p)**	**M(SD)**	**M(SD)**	**t(p)**	**t(p)**	**t(p)**
**GHQ-28**								
Total score	6.22 (4.45)	9.84 (6.02)	2.241 (0.031^*^)	6.37 (4.99)	7.67 (5.87)	1.060 (0.292)	0.125 (0.901)	−1.296 (0.201)
Physical symptoms	2.91 (2.17)	3.74 (2.60)	1.119 (0.270)	2.58 (2.30)	2.81 (2.19)	0.441 (0.661)	−0.569 (0.572)	−1.406 (0.166)
Insomnia and Anxiety	1.57 (1.47)	3.53 (2.14)	3.504 (0.001^*^)	2.09 (2.03)	2.28 (1.98)	0.407 (0.685)	1.099 (0.276)	−2.161 (0.035^*^)
Social activity	1.43 (1.93)	1.89 (1.88)	0.778 (0.441)	1.28 (1.71)	1.56 (2.03)	0.657 (0.513)	−0.337 (0.737)	−0.603 (0.549)
Depression	0.17 (0.49)	0.68 (1.11)	1.862 (0.075)	0.40 (0.88)	0.97 (1.80)	1.760 (0.085)	1.315 (0.193)	0.733 (0.467)
**STAI**								
State–anxiety	2.57 (0.79)	3.53 (0.77)	3.970 (*p* < 0.001^*^)	2.77 (0.90)	2.75 (1.16)	−0.076 (0.940)	0.910 (0.366)	−2.629 (0.011^*^)
Trait–anxiety	2.13 (1.01)	3.21 (1.08)	3.331 (0.002^*^)	2.42 (0.98)	2.78 (1.24)	1.434 (0.156)	1.124 (0.265)	−1.280 (0.206)

*GHQ-28, General Health Questionnaire-28; STAI, State–Trait Anxiety Inventory; SD, standard deviation;*

* *p < 0.05*.

a *T1: First wave in Japan, from the beginning of March to the end of May 2020*.

b *T2: Secondary wave in Japan, from the beginning of June to the end of August 2020*.

Similarly, state–anxiety (t = 3.970, *p* < 0.001) and trait–anxiety (t = 3.331, *p* = 0.002) were higher in female than male participants at T1, but no gender difference was found in these scales at T2 (t = −0.076, *p* = 0.940 and t = 1.434, *p* = 0.156, respectively). We found that anxiety/insomnia (t = −2.161, *p* = 0.035) and state–anxiety (t = −2.629, *p* = 0.011) were significantly lower at T2 than T1 in female participants. On the other hand, there was no significant difference in male participants regarding these items (t = 1.099, *p* = 0.276, and t = 0.910, *p* = 0.366, respectively).

Due to the presence of data that were not normally distributed or not homogeneous in their variance, non-parametric tests were also conducted to confirm the results of MANCOVA analysis. The results of Mann-Whitney *U*-test were essentially the same as those by MANCOVA analysis (Supplementary Table 1).

## Discussion

In this study, we examined the mental health problems of hospitalized COVID-19 patients, focusing on depression and anxiety, during the primary and secondary wave of the pandemic. It is not clear whether anxiety and depression were higher in each subject than before the pandemic with specificity for COVID-19 since there was no comparative reference data of the subjects before COVID-19. However, it would be worthwhile to present the basic data for conducting a longitudinal survey regarding the effects of COVID-19 infection on mental health in the future, since this pandemic has not been completely converged. The results of this study revealed that women exhibited more anxiety than men did during the primary wave of COVID-19. However, this gender difference disappeared during the secondary wave of infections, with female anxiety presenting less often. The average scores of the GHQ-28 and STAI at T2 for men were higher than those at T1, although this difference was not statistically significant. These findings were partially unexpected given our initial hypothesis; women had to deal with more mental health problems at both time points. Thus, mental health care during a pandemic should be differentiated by gender, and the differences in mental health between the primary and secondary waves of the event should be taken into consideration.

The literature already established that women exhibit anxiety more than men (8). They are also more likely to seek social support, often employing emotion-focused coping strategies. However, men take a more problem-focused approach (17). The high-anxiety trait in women could be responsible for their high scores for anxiety/insomnia, state–anxiety, and trait–anxiety at T1 in our study.

In the secondary wave, a greater prevalence of information on the pandemic may have reduced patient anxiety. The improvement in the anxiety/insomnia and state–anxiety scores for women may be attributed to their emotion-focused skills, such as better interpersonal communication abilities (18–20). This may have benefited them during recovery.

Some studies showed that men are more emotionally inhibited than women (21). One possible interpretation for the absence of significant improvement of the GHQ-28 and STAI scores in male subjects might be due to the lack of communication in male patients, which could have contributed to greater anxiety. Although the quantity of information on the virus and disease increased at T2, it has been suggested that COVID-19-related mental health problems such as suicides (22) and the complex grief of bereaved families may increase (23). The course of the pandemic is still unpredictable. We thus attribute the lack of improvement in men's GHQ-28 and STAI scores at T2 to the inability to apply problem-focused strategies to cope with the pandemic, although the direct assessment of communication skills would be considered in a further follow-up survey.

To summarize, female patients with COVID-19 were more anxious during the primary wave of the pandemic, whereas male patients might have had difficulties in coping with anxiety, considering the lack of improvement in their mental health over time. We concluded that women may need more care in the early phase of a crisis, whereas men might need care during the follow-up period. We do reiterate that thorough mental care for all COVID-19 patients is important, regardless of gender.

This study has several limitations. First, we could not compare the results of the same patient sample based on their hospitalization periods. Strictly speaking, the design of the current study is a repeated cross-sectional one at two-time points. Therefore, the results of the current study should be interpreted with caution. A longitudinal follow-up study on the same participants would be needed to investigate the changes in their psychological status over time. Second, factors such as socioeconomic ones which potentially influence mental status could not be considered because of the lack of substantial information in the current study. Third, patients with moderate to severe COVID-19 infection could not complete the questionnaire owing to their ill-health. Fourth, the sample size was relatively small. We believe that assessing severe cases with larger sample sizes, for example, by conducting a multi-institutional joint research in a longitudinal follow-up design, can be a fruitful endeavor to understanding care during a pandemic. Finally, only subjective measures were included in this study. Future studies should obtain objective and subjective data to improve the validity of the survey.

## Conclusion

Female participants with COVID-19 infection were more anxious during the early phase of the pandemic, whereas both genders showed similar attitudes in the secondary wave, with female anxiety presenting less often than those in the first wave. Gender-specific mental care is needed, particularly for women at an early stage of an epidemic.

## Data Availability Statement

The data analyzed in this study is subject to the following licenses/restrictions: data usage for secondary purpose has not been approved by Institutional Review Board of Amagasaki General Medical Center. Requests to access these datasets should be directed to Hironobu Fujiwara, hirofuji@kuhp.kyoto-u.ac.

## Ethics Statement

The studies involving human participants were reviewed and approved by the Hyogo Prefectural Amagasaki General Medical Center Institutional Review Board. Written informed consent for participation was not required for this study in accordance with the national legislation and the institutional requirements.

## Author Contributions

RT, HF, YK, NI, MS, KM, and HM conceived and designed the study. RT and HF contributed to the data analysis and interpretation, along with the drafting of the manuscript. YK contributed to the drafting of the manuscript. KM contributed to the revision of the critical analysis. RT, NI, and MS contributed to the data acquisition. All authors approved the final manuscript for submission and agreed to be accountable for all aspects of the work, including the assurance that questions related to the accuracy or integrity of any part are appropriately investigated.

## Funding

This study was supported by a management expenses grant from the Hyogo Prefectural Amagasaki General Medical Center and a Grant-in-Aid for Scientific Research (B) (Japan Society for The Promotion of Science, 21H02849).

## Conflict of Interest

The authors declare that the research was conducted in the absence of any commercial or financial relationships that could be construed as a potential conflict of interest.

## Publisher's Note

All claims expressed in this article are solely those of the authors and do not necessarily represent those of their affiliated organizations, or those of the publisher, the editors and the reviewers. Any product that may be evaluated in this article, or claim that may be made by its manufacturer, is not guaranteed or endorsed by the publisher.
