# Supplementary material for: Gender Differences in Anxiety Among COVID-19 Inpatients Under Isolation: A Questionnaire Survey During the First and Second Waves of the COVID-19 Pandemic in Japan
Source: Front Public Health. 2021 Oct 20;9:708965. doi: 10.3389/fpubh.2021.708965 (PMC8563575; doi:10.3389/fpubh.2021.708965)
Supplement: Supplementary file 1 [file Table_1.docx]

Supplementary table. The main results of non-parametric analyses.

|  | T1 | T2 | Mann-Whitney U tests between  T1 and T2 in each gender | |
| --- | --- | --- | --- | --- |
|  | Mann-Whitney U tests  Male/Female (p) | Mann-Whitney U tests  Male/Female (p) | Male (p) | Female (p) |
| GHQ-28 |  |  |  |  |
| Total score | 0.049* | 0.424 | 0.834 | 0.186 |
| Physical symptoms | 0.296 | 0.629 | 0.483 | 0.177 |
| Insomnia and anxiety | 0.003* | 0.681 | 0.424 | 0.031* |
| Social activity | 0.307 | 0.754 | 0.842 | 0.337 |
| Depression | 0.062 | 0.398 | 0.308 | 0.803 |
| STAI |  |  |  |  |
| State-Anxiety | 0.001* | 0.951 | 0.403 | 0.014* |
| Trait-Anxiety | 0.003* | 0.129 | 0.295 | 0.251 |

GHQ-28, General Health Questionnaire-28; STAI, State–Trait Anxiety Inventory; *p <0.05
